# Naloxone dosing in the era of synthetic opioids: Applying the Goldilocks principle

**DOI:** 10.1111/add.70060

**Published:** 2025-04-08

**Authors:** Mariana Gonzalez Utrilla, Edward Chesney, Joanne Neale, Nicola Metrebian, Nicola Kalk, Arne Kristian Skulberg, Paul Dietze, Martin Smith, John Strang

**Affiliations:** ^1^ National Addiction Centre Institute of Psychiatry, Psychology & Neuroscience, King’s College London London UK; ^2^ South London and Maudsley NHS Foundation Trust London UK; ^3^ Centre for Social Research in Health University of New South Wales Sydney New South Wales Australia; ^4^ Division of Prehospital Services Oslo University Hospital Oslo Norway; ^5^ Department of Circulation and Medical Imaging Norwegian University of Science and Technology Trondheim Norway; ^6^ Disease Elimination Program Burnet Institute Melbourne Australia; ^7^ National Drug Research Institute and Enable Institute Curtin University Melbourne Australia; ^8^ Derbyshire Healthcare NHS Foundation Trust Derby UK

**Keywords:** naloxone, naloxone dosage, opioid antagonist, opioid overdose, opioid overdose reversal, overdose prevention

## Abstract

The opioid overdose epidemic remains a critical public health crisis. In recent years, synthetic opioids like fentanyl and nitazenes, have driven a sharp rise in deaths. Naloxone, an opioid receptor antagonist, has been established as a key intervention for reversing opioid overdoses and saving lives. However, there remains a critical need to optimize naloxone dosing strategies. This article examines the challenges of both under‐dosing and over‐antagonism in naloxone administration, emphasizing the importance of a tailored approach to overdose management. A ‘just the right dose’ approach is essential for minimizing the risks of over‐antagonism while still reversing opioid overdose. This involves starting with a modest naloxone dose and carefully titrating it based on the patient's response, considering factors such as opioid type, overdose severity, and opioid tolerance. A tailored approach to naloxone dosing may present challenges for non‐medically trained responders but it can ensure that as many people as possible receive the right dose of naloxone when they need it. Training programs should also emphasize the importance of first aid and supportive care, including airway management and alerting emergency services, as well as careful monitoring of the patient's response.

## INTRODUCTION

The opioid overdose epidemic remains a critical public health crisis, with synthetic opioids like fentanyl and nitazenes driving a sharp rise in deaths. In 2022, the US Centers for Disease Control and Prevention (CDC) reported 107 941 drug overdose deaths in the USA, with 73 838 (70%) involving synthetic opioids [1]. Between 2016 and 2021, synthetic opioid‐related deaths quadrupled, increasing from 5.7 to 21.6 per 100 000 [2, 3]. In Europe, 6392 overdose deaths were reported in 2022, with opioids involved in 74% of cases [4]. The UK recorded 4859 drug‐related deaths in England and Wales in 2022, the highest since records began, while Scotland reported 1051 deaths, maintaining Europe’s highest drug death rate [5, 6]. In Australia, 1819 drug‐induced deaths occurred in 2022, equating to five deaths per day, with opioids involved in 62% (1123 deaths) [7].

Patterns of polysubstance use, including opioids combined with benzodiazepines and alcohol, alongside novel synthetic opioids, have prompted calls for an urgent need for adaptive strategies in overdose monitoring, prevention and treatment, including a re‐evaluation of naloxone dosing protocols and the development of more effective overdose reversal strategies [8].

Naloxone, a μ‐opioid receptor antagonist, remains the primary treatment for reversing opioid overdoses. It was traditionally administered intravenously or intramuscularly at a recommended initial dose of 0.4 mg, with newer intranasal formulations developed to improve acceptability for non‐medical responders and to encourage bystander carriage rate [9, 10, 11]. Nyxoid® and Ventizolve® are intranasal formulations delivering doses roughly equivalent to a 0.4 mg intramuscular injection, with 1.8 mg and 1.26 mg doses, respectively [12, 13]. Intramuscular naloxone typically acts within 2–5 minutes, while intranasal formulations may have a slightly slower onset of 3–7 minutes owing to nasal mucosal absorption [14].

The rise of highly potent synthetic opioids has prompted a re‐evaluation of traditional naloxone dosing regimens. Fentanyl is 50–100 times more potent than morphine, while its analogue carfentanil is approximately 10 000 times more potent [15]. Emerging nitazene opioids have a potency similar to fentanyl and also produce active metabolites [16]. This increased potency raises concerns that standard naloxone doses may be inadequate for reversing overdoses effectively, particularly in cases involving these synthetic opioids [17].

In response to these challenges, new naloxone formulations have been developed, after Narcan® (4 mg) was the first to receive approval in 2015, followed by the development of other higher‐dose intranasal sprays and intramuscular autoinjectors (Table 1). These products, such as the 8 mg Kloxxado™ nasal spray and the 5 mg/0.5 ml ZIMHI™ intramuscular injection, aim to address the increased potency of synthetic opioids and reduce the need for multiple administrations [18, 19]. However, their introduction raises important questions about optimal dosing strategies and potential risks associated with higher naloxone doses, especially when they are intended to be administered by non‐medical responders.

**TABLE 1:** Different naloxone products (2022–2024).

Commercial name	Route of administration	Dose (as labelled)	Equivalent dose as naloxone hydrochloride	Number of doses per package/total available
Narcan®	Intranasal	4 mg/0.1 ml	4 mg	2 single‐dose containers per package (8 mg total)
Kloxxado™		8 mg/0.1 ml	8 mg	2 single‐dose containers per package (16 mg total)
Nyxoid®		1.8 mg/0.1 ml	2 mg	2 single‐dose containers per package (4 mg total)
Ventizolve®		1.26 mg/0.1 ml	1.4 mg	2 single‐dose containers per package (2.8 mg total)
RiVive®		3 mg/0.1 ml	3 mg	2 single‐dose containers per package (6 mg total)
Rezenopy®		10 mg/0.1 ml	10 mg	2 single‐dose containers per package (20 mg total)
ZIMHI®	Intramuscular	5 mg/0.5 ml	5 mg	One single‐dose, prefilled syringe (5 mg total)
Prenoxad®		0.4 ml/ml	2 mg	5 doses of 0.4 mg of one pre‐filled syringe (2 mg total)
Glass ampoule		0.4 mg/ml	0.4 mg/ml	–

The question of appropriate naloxone dosing is critical for several reasons. With the increasing prevalence of potent synthetic opioids, there is a pressing need to ensure that the naloxone doses administered are sufficient to restore independent breathing and prevent cardiac arrest or death, particularly in the crucial period before full medical care arrives [20]. This clinical efficacy must be balanced against patient experience, as naloxone can significantly impact the individual, potentially causing severe withdrawal symptoms and making post‐overdose care difficult if too high a dose is given [21]. Excessive dosing may precipitate aggression or violence, and in some cases, could trigger increased drug‐seeking behaviour, potentially exacerbating the overdose problem through subsequent self‐administration of additional opioids to counteract the withdrawal effects.

Naloxone dosing has entered a paradoxical phase. In North America, nasal devices for non‐medically trained responders are available in twin packs containing either 4 mg (Narcan®) or 8 mg (Kloxxado™), with proposals for doses as high as 12 mg [19, 22]. In contrast, Europe and Australia use significantly lower‐dose nasal sprays, with twin packs containing 1.8 mg (Nyxoid®) or 1.4 mg (Ventizolve®) [13, 23].

As we navigate this evolving overdose crisis, finding the right balance between effective overdose reversal and minimizing adverse effects remains a critical challenge in our efforts to save lives and mitigate the impact of the opioid epidemic.

### Not enough is not enough

The pharmacological properties of fentanyl and other synthetic opioids might present a compelling case for higher naloxone dosages in opioid‐associated emergencies. The potency and lipophilicity of fentanyl lead to the rapid onset of strong central nervous system (CNS) effects [24]. This creates a scenario where standard naloxone doses may be insufficient to compete effectively with μ‐opioid receptor binding sites [25]. Multiple studies have documented cases where several standard doses of naloxone were required to reverse synthetic opioid overdoses, with some needing up to 8 mg or more [26].

Greater penetration into the CNS, combined with the shorter duration of action of naloxone, also means that overdose symptoms may re‐emerge once naloxone wears off [26]. Higher or repeated doses of naloxone may be required to sustain therapeutic levels for a longer duration, potentially mitigating the risk of renarcotisation [21].

Moreover, fentanyl and its analogues can induce a potentially life‐threatening condition known as wooden chest syndrome or fentanyl‐induced respiratory muscle rigidity [27]. This phenomenon, characterized by severe muscle rigidity and laryngospasm, can occur within minutes of fentanyl injection and may complicate overdose management [28]. Wooden chest syndrome primarily impairs ventilation by causing intercostal muscle contraction that restricts chest wall expansion, which might require higher doses of naloxone for effective reversal [24, 27].

Moss *et al*. [29] developed a theoretical model predicting that higher naloxone doses may be needed to effectively reverse fentanyl overdoses. Their model suggested that a 2 mg intramuscular dose might be insufficient to reverse high‐exposure fentanyl overdoses within 10 minutes. However, while these models are increasingly sophisticated, they remain theoretical and may not fully reflect the complexities of real‐world clinical scenarios. Such models should complement, not replace, evidence from controlled clinical trials and real‐world patient care when establishing optimal dosing strategies [30].

A retrospective study by Carpenter *et al*. [31] found that the median total naloxone dose administered intravenously by emergency medical services (EMS) for suspected fentanyl overdose before hospital arrival was 0.8 mg (interquartile range [IQR] = 0.4–1.38 mg). However, a quarter of patients required higher doses (≥1.2 mg i.v. equivalent), with one reaching up to 5.2 mg. Notably, the study showed that many patients, even in suspected fentanyl overdoses, responded effectively to lower doses. Additionally, there were no significant differences in naloxone doses required to reverse fentanyl versus non‐fentanyl opioid overdoses. This variability in doses is not unique to the synthetic opioid era; Cantwell *et al*. [32] demonstrated similar variations in naloxone requirements for suspected heroin overdoses, influenced by a range of variables including concomitant substance use.

Similarly, Bell and colleagues questioned the extent to which higher naloxone doses were really required or given in practice [33]. They investigated 1072 overdose episodes between 2013 and 2016 and found no statistically significant changes in the number of doses of naloxone given over time [23]. Later studies on synthetic opioids including fentanyl show only modest increases in naloxone doses required by ambulance paramedics [34].

Somerville *et al*. [35] provided real‐world evidence from a mixed‐methods study indicating that 83% of 64 participants who either witnessed or experienced an opioid overdose reported that two or more doses of naloxone (usually intranasal 2 mg/ml) were required before the individual showed a response to a suspected fentanyl overdose. Similarly, a study by Amaducci *et al*. [17] found that patients with novel potent opioid (NPO) overdoses required a higher number of in‐hospital naloxone boluses compared with those with fentanyl overdoses. However, the sample size of this study was very small (with *n* = 9 in the NPO group and *n* = 11 in the fentanyl group) [17].

### Too much is too much

While higher doses of naloxone may occasionally be necessary to address some synthetic opioid overdoses, there is significant evidence supporting the effectiveness of smaller doses [36]. Moreover, high doses carry the risk of over‐antagonism, which can lead to a range of adverse outcomes.

In 2015, Neale & Strang first introduced the concept of ‘over‐antagonism’, where excessive naloxone dosing could lead to problematic outcomes, including acute withdrawal symptoms, aggressive behaviour, refusal of further medical care and premature self‐discharge [37]. Strang *et al*. [38] expanded on the concept, proposing various forms of ‘toxicity’ associated with naloxone use: pharmacological, physiological, behavioural and reputational.

Pharmacological toxicity refers to the severe withdrawal symptoms precipitated by rapid opioid reversal [39, 40]. A comprehensive review by Pergolizzi *et al*. [39] highlighted the potential for naloxone‐induced withdrawal symptoms that not only cause immediate distress but may also negatively impact future healthcare‐seeking behaviours among individuals who use opioids. A study by Payne *et al*. [40] provided real‐world data comparing 8 mg with 4 mg intranasal naloxone, finding that recipients of 8 mg intranasal naloxone had 2.5 times the risk of withdrawal symptoms, though no significant differences in survival rates were observed.

Physiological toxicity, particularly pulmonary effects, has also been a focus of recent research on naloxone administration. A retrospective analysis by Farkas *et al*. [41] found a significantly higher risk of pulmonary complications (42% vs 26%) in patients receiving out‐of‐hospital naloxone doses exceeding 4.4 mg, compared with lower doses. Kummer *et al*. [42] reported 10 severe cases of naloxone‐associated pulmonary oedema, with a median naloxone dose of 4.25 mg and with doses reaching up to 19 mg in some patients. These findings highlight the importance of careful respiratory monitoring post‐reversal [41, 42]. Further research is needed to clarify the relationship between naloxone dosing and pulmonary complications, as factors such as overdose severity may influence both naloxone use and complication rates.

The concept of behavioural toxicity includes problematic behaviours caused by naloxone over‐antagonism. Neale & Stang [37] highlighted this in a qualitative study where people using heroin reported negative experiences with naloxone, resulting in aggression towards hospital staff and premature self‐discharge. Ferguson *et al*. [43] further explored the experience of being ‘narcanned’ and its potential to trigger conflict. Quantitative data from Scheuermeyer *et al*. [44] involving 1009 presumed fentanyl overdoses found 10.4% of patients left before physician assessment, and 14.3% left against medical advice after assessment. Neale *et al*. [45] found that people who had overdosed were significantly more likely to display anger if the person resuscitating them criticised, berated or chastised them during resuscitation, suggesting that improved training in overdose response could reduce adverse reactions.

Reputational toxicity describes the reluctance of overdose victims and bystanders to use naloxone, particularly high‐dose formulations, owing to fears of hostile reactions or unpleasant outcomes, and may also discourage people from calling emergency services. Bessen *et al*. [46] conducted a qualitative study demonstrating resistance to carrying or using naloxone, as negative experiences with aggressive or violent behaviour following naloxone administration discouraged individuals from using this treatment, potentially weakening community‐based overdose response efforts. Strickland *et al*. [47] surveyed patient perceptions of higher‐dose naloxone nasal sprays, finding that while 36% preferred higher doses, 11% expressed concerns about severe withdrawal symptoms.

### Just the right dose is just the right dose

Supportive care, including physical stimulation and basic airway management, is critical before naloxone administration. Dietze *et al*. [48] found that 90% of overdose events at a supervised injection site were resolved with airway management alone. When naloxone was administered, an intramuscular dose was more effective, requiring fewer additional doses compared with the same dose administered intranasally (8.6% vs 23.1%).

As we address the ongoing opioid crisis, balancing effective overdose reversal with minimizing negative outcomes from over‐antagonism remains key to developing successful strategies for opioid overdose management. Given the complexities of both under‐dosing and over‐antagonism in naloxone administration, the ideal of administering ‘just the right dose’, enough to reverse the overdose but never more than required, has long been recognized [36, 49, 50]. This method involves the initial administration of a modest naloxone dose followed by careful assessment and additional doses as required, akin to the tailored use of antagonists during anaesthesia recovery [11].

Naloxone dosing for opioid overdose reversal varies based on factors like drug dose, use behaviours, opioid tolerance, and age [32, 51]. Evidence shows that low‐dose intranasal naloxone can be effective. Kelly *et al*. [52] reported that 74% of likely heroin overdoses were reversed within 8 minutes using a low‐concentration intranasal preparation (2 mg/5 ml) in a prehospital EMS setting, though significant dose wastage was noted due to the nose's limited capacity to absorb medication, with much of the solution running out of the nostrils or down the throat before it could be properly absorbed into the bloodstream. A more concentrated preparation (2 mg/1 ml) achieved a similar response rate (82%), but wastage remained an issue [53]. Dietze *et al*. [48] found that intranasal naloxone (800 μg/1 ml) could reverse opioid overdose at the Sydney Medically Supervised Injecting Centre, but was less effective than intramuscular administration, with more patients requiring rescue doses (23.1% vs 8.6%) and longer times to adequate response. Skulberg *et al*. [54] found that intramuscular naloxone (0.8 mg/2 ml) was more successful (97.2%) at restoring breathing within 10 minutes compared with low‐dose intranasal naloxone (1.4 mg/0.1 ml, 79.6%), with fewer patients needing additional doses (9.3% vs 29.0%).

Cho *et al*. [55] examined naloxone administration at Insite, a supervised injection facility in Vancouver. Dosing followed the BC Centre for Disease Control clinical decision support tool, starting with 0.4 mg (or 0.4–0.8 mg for muscle rigidity), with additional 0.4 mg doses every 2–3 minutes until the respiratory rate exceeded 10 breaths per minute. In 2014–2015, 73.9% of patients with confirmed reversal required one dose, 22.2% needed two doses and 4.0% required three. These proportions remained similar in 2016–2017 (74.2%, 22.7% and 3.0%), despite a rise in overdose events from 586 to 2033 and a decrease in successful reversals. Reversal rates dropped from 31.4% in 2014 to 22.9% in 2017. This suggests that while more potent opioids like fentanyl have increased overdose challenges, they have not necessitated higher naloxone doses. It highlights the importance of first aid, careful titration and the risks of over‐antagonism with excessive dosing.

To effectively implement this approach in real‐world scenarios, several key elements need to be considered. The initial dose should be sufficient to improve respiratory function without causing severe withdrawal [50]. This ‘modest’ dose may vary depending on factors such as the suspected opioid involved, the patient’s opioid tolerance, the concomitant consumption of other CNS depressant drugs and the severity of the overdose. Current research suggests that an initial dose of 0.4–0.8 mg (i.m./i.v. equivalent) may be appropriate in many cases, but that much higher doses may be needed in some overdose emergency situations. Doses will likely need to be tailored based on the specific situation [32, 51, 56].

### Implementing a tailored approach

The real challenge lies in applying the principle of naloxone administration effectively for non‐medically trained individuals under pressure. Striking a balance between effective overdose reversal and minimizing adverse effects is vital, yet it presents unique difficulties in community settings. Naloxone should not be the first intervention in opioid emergencies; basic first aid—such as clearing the airway, physical stimulation, or cardiopulmonary resuscitation (CPR)—is essential [57]. Training for both medical and non‐medical responders, along with policy development, must prioritize this chain of care. Ignoring these steps risks disconnecting science and policy from the real‐world treatment of patients with reduced consciousness and abnormal breathing.

To enable tailored dosing, especially by non‐medical individuals, attention must be given to alerting emergency services, providing first aid and using incremental dosing. After the initial dose careful monitoring of the patient’s response is needed, focusing on respiratory rate, consciousness, pupil size and heart rate. Frequent reassessment is essential, with additional doses administered as needed to reverse respiratory depression without causing severe withdrawal. Smaller doses (e.g. 0.1–0.2 mg) can be given every 2–3 minutes until the desired effect is achieved [21].

Given the potential for re‐sedation with the longer half‐life of many opioids compared with naloxone, ongoing monitoring of the person who overdosed is essential. This is particularly important in cases involving long‐acting opioids. Factors such as the individual’s opioid tolerance, the specific opioid involved and any co‐ingested substances should be taken into account.

This approach to naloxone administration has several advantages. Gradual opioid reversal may enhance patient comfort and improve cooperation with further treatment, reducing the likelihood of patients leaving against medical advice and allowing for more comprehensive care. Minimizing severe withdrawal symptoms could lower the risk of agitation or aggression, improving safety for both patients and healthcare providers. Additionally, this method is adaptable to diverse overdose scenarios, accommodating cases requiring minimal intervention as well as severe overdoses needing higher total doses.

### Beyond naloxone: a role for nalmefene?

Nalmefene, which has a longer half‐life and higher affinity for the opioid receptor than naloxone, has been proposed as an alternative overdose reversal agent. Two studies in healthy volunteers compared the effects of single doses of nalmefene with naloxone on opioid‐induced respiratory depression [58, 59]. In a study by Cipriano and colleagues an intramuscular dose of nalmefene 1 mg outperformed intranasal naloxone 4 mg in reversing respiratory depression within 5 minutes but was no more effective than intramuscular naloxone 2 mg [59]. A study by Ellison and colleagues compared intranasal nalmefene 2.7 mg with intranasal naloxone 4 mg [58]. At 5 minutes, the nalmefene had had a greater impact on respiratory rate.

While the longer half‐life of nalmefene (10 vs 1.5 hours) may reduce the risks of respiratory depression re‐emerging, it could also result in sustained opioid withdrawal symptoms, possibly deterring those receiving it from seeking future emergency care [60]. Similarly, while the higher affinity of nalmefene for the receptor may contribute to its rapid effects, this also increases the risk of people experiencing severe withdrawal symptoms. Ultimately, we will need more data from controlled studies in patient populations as well as real‐world outcome data before these findings can be translated into clinical practice.

### The role of the pharmaceutical industry

It has been noted that many of the most vocal advocates for higher naloxone dosing appear to be associated with the pharmaceutical industry. They have heavily promoted higher doses and new delivery systems, often presenting data that may be influenced by vested financial interests [61]. High‐dose naloxone formulations, which have been marketed as necessary to combat potent synthetic opioids, despite the limited evidence supporting this hypothesis, can cost up to 10 times more than a generic formulation, driving up healthcare costs significantly [36]. Whereas existing research indicates that even with the increasing prevalence of fentanyl‐related overdoses, standard naloxone doses continue to demonstrate effectiveness in numerous situations.

### Implications and future directions

The ‘just right dose’ approach offers significant benefits but also comes with some challenges [50]. Effective implementation requires additional training and close monitoring for both medical professionals and non‐medical responders. The urgency to reverse an overdose may conflict with the time needed for careful dose titration, which can be more resource‐intensive than a single large dose. However, most injectable and intranasal naloxone formulations allow for some degree of dose adjustment based on patient response. While titrated dosing can be challenging in high‐stress emergencies, recent research, such as Madah‐Amiri’s study in Oslo, has shown that non‐medical responders can successfully apply this approach in community settings [57, 62].

Future research and clinical practice should further explore naloxone over‐antagonism and emphasize post‐reversal interventions. The period after overdose reversal offers a potential ‘teachable moment’ to promote harm reduction and long‐term recovery [11, 63, 64, 65]. However, its effectiveness varies, as it may benefit some while alienating others if not approached thoughtfully. Further research is needed to understand the complexities of post‐overdose care and to develop person‐centred strategies that address the diverse needs of individuals who use drugs.

The debate between low‐dose and high‐dose naloxone approaches highlights the need for randomized controlled trials (RCTs) to resolve clinical equipoise. Pharmacodynamic studies and real‐world data on low‐dose strategies could clarify their balance of efficacy and safety, particularly for synthetic opioid overdoses. Comparing intramuscular and intranasal administration may also uncover differences in onset time, success rates and repeat dosing requirements. A cluster RCT with ambulance services could evaluate tailored strategies that balance rapid overdose reversal with minimizing adverse effects like severe withdrawal. Further data collection within a range of environments such as emergency departments, ambulances, drug consumption rooms and outreach services would be helpful in informing future community‐based interventions.

## CONCLUSION

Overdose prevention and management is at a pivotal point. Effective basic first aid, including airway management, is essential before naloxone administration, with ambulance call‐out remaining critical. While high‐dose naloxone protocols and take‐home naloxone programs expand, the professional medical response remains indispensable. Training for take‐home naloxone should emphasize interim emergency care until medical personnel arrive. It is vital to ensure that increased access to naloxone for non‐medical responders does not unintentionally reduce ambulance attendance, as this could inadvertently increase harm.

The future of naloxone administration lies in personalized dosing, requiring a detailed understanding of dose titration. This shift towards more personalized dosing will necessitate enhanced training programs for both professionals and non‐medically trained responders, covering not only naloxone administration but also post‐reversal care and interventions.

To meet these evolving needs, naloxone products and kits will need to become more dose flexible. While many current products already come in twin packs or larger quantities, future developments should explore wider multi‐dose formats of nasal sprays, injectable pre‐filled syringes or other novel formulations.

By addressing these factors and conducting rigorous research, we can develop more effective strategies for overdose prevention and post‐overdose care. This approach must balance immediate life‐saving interventions with long‐term patient wellbeing and public health considerations. As the opioid crisis evolves, strategies must remain adaptable, evidence‐based and focused on the needs of those at risk. Through ongoing research, innovation and collaboration with people who use drugs, those with lived experience and all sectors of healthcare, we can hope to make meaningful progress in reducing the devastating impact of opioid overdoses.

## AUTHOR CONTRIBUTIONS

**Mariana Gonzalez Utrilla:** Conceptualization (equal); methodology (equal); writing — original draft (equal); writing — review and editing (equal). **Edward Chesney:** Conceptualization (equal); methodology (equal); writing — original draft (equal); writing — review and editing (equal). **Joanne Neale:** Conceptualization (equal); methodology (equal); writing — review and editing (equal). **Nicola Metrebian:** Conceptualization (equal); methodology (equal); writing — review and editing (equal). **Nicola Kalk:** Conceptualization (equal); methodology (equal); writing — review and editing (equal). **Arne Kristian Skulberg:** Conceptualization (equal); methodology (equal); writing — review and editing (equal). **Paul Dietze:** Conceptualization (equal); methodology (equal); writing — review and editing (equal). **Martin Smith:** Conceptualization (equal); methodology (equal); writing — review and editing (equal). **John Strang:** Conceptualization (equal); methodology (equal); writing — original draft (equal); writing — review and editing (equal).

## DECLARATION OF INTERESTS

M.G.U., E.C., P.D., N.K. and M.S. have no interests to declare. A.K.S. has received two speaker fees (one in 2023 and one in 2024) from Accord Ltd UK, which sells naloxone 1.26 mg/dose in the UK. A.K.S. has no other financial or in‐kind benefits from the sale of any naloxone product. In the last 3 years, J.N. has secured, through her university, research funding from Mundipharma Research Ltd to study the effectiveness of naloxone. Mundipharma developed and market the concentrated naloxone nasal spray, Nyxoid®. J.N. has also secured, through her university, funding from Camurus AB and honoraria from Indivior and Camurus AB for research and presentations unrelated to naloxone. In the last 3 years, N.M. has received, through her university, King’s College London, research funding from Mundipharma Research Ltd, a pharmaceutical company that produces a naloxone nasal spray. Through his employer (King’s College London), J.S. has worked with pharmaceutical and technology companies that have supported the university with grants and/or honoraria and/or consultancy payments, as described at https://www.kcl.ac.uk/people/john-strang (including, in the past 3 years, MundiPharma, Camurus, Pneumowave, Accord and dne), and have also supplied medications or devices (Pneumowave, CMI and Catalent) to develop or study potentially improved formulations and devices. His employer (King’s College London) previously registered intellectual property on an innovative buccal naloxone treatment with which J.S. is involved, and he has previously been named in a patent registration by a Pharma company as the inventor of a concentrated naloxone nasal spray. He is also named as a Patron of Addiction Family Support (formerly DrugFAM), a UK‐based registered charity.

## Data Availability

Data sharing not applicable to this article as no datasets were generated or analysed during the current study.

## References

[1] FB A, JA C, LM R, P S. National Center for Health Statistics . Provisional drug overdose death counts. 2024 Available from: https://www.cdc.gov/nchs/nvss/vsrr/drug-overdose-data.htm#drug_specificity_1

[2] Brian Tsai. National Center for Health Statistics . Fentanyl Overdose Death Rates More Than Tripled From 2016 to 2021. 2023 Available from: https://blogs.cdc.gov/nchs/2023/05/03/7338/

[3] Spencer MR , Warner M , Cisewski JA , Miniño A , Dodds D , Perera J , et al. Estimates of Drug Overdose Deaths Involving Fentanyl, Methamphetamine, Cocaine, Heroin, and Oxycodone: United States, 2021. 2023;(27):1–14. Available from: https://www.cdc.gov/nchs/products/index.htm

[4] European Monitoring Centre for Drugs and Drug Addiction . European Drug Report 2023: Trends and Developments 2024; 2023.

[5] Office for National Statistics (ONS). Deaths related to drug poisoning in England and Wales: 2022 registrations. ONS Website [Internet] 2023. Available from: https://www.ons.gov.uk/peoplepopulationandcommunity/birthsdeathsandmarriages/deaths/bulletins/deathsrelatedtodrugpoisoninginenglandandwales/2021registrations

[6] National Records of Scotland. Drug‐related Deaths in Scotland in 2022. 2023;(August).

[7] Chrzanowska A , Man N , Sutherland R , Degenhardt L , Peacock A . Overdose drug‐induced deaths other drug‐induced deaths. Nat Drug Alcohol Res Centre. 2024;2003–2022.

[8] Jones JD , Mogali S , Comer SD . Polydrug abuse: a review of opioid and benzodiazepine combination use. Drug Alcohol Depend. 2012;125(1–2):8–18. 10.1016/j.drugalcdep.2012.07.004 22857878 PMC3454351

[9] Kim HK , Nelson LS . Reducing the harm of opioid overdose with the safe use of naloxone: a pharmacologic review. Expert Opin Drug Saf. 2015;14(7):1137–1146. 10.1517/14740338.2015.1037274 25865597

[10] Krieter PA , Chiang CN , Gyaw S , McCann DJ . Comparison of the pharmacokinetic properties of naloxone following the use of FDA‐approved intranasal and intramuscular devices versus a common improvised nasal naloxone device. J Clin Pharmacol. 2019;59(8):1078–1084. 10.1002/jcph.1401 30861160 PMC6606390

[11] WHO . Community management of opioid overdose World Health Organization; 2014. 25577941

[12] (EMA) EMA . Nyxoid, Naloxone hydrochloride dihydrate. European medicines agency science medicine Health 2023;

[13] AS DP . Ventizolve summary of product characteristics. Pharmaceut Med. 2019:1–7.31933270

[14] Saari TI , Strang J , Dale O . Clinical pharmacokinetics and pharmacodynamics of naloxone. Clin Pharmacokinet. 2024;63(4):397–422. 10.1007/s40262-024-01355-6 38485851 PMC11052794

[15] Concheiro M , Chesser R , Pardi J , Cooper G . Postmortem toxicology of new synthetic opioids. Front Pharmacol. 2018;9(OCT):1–18. 10.3389/fphar.2018.01210 30416445 PMC6212520

[16] Pucci M , Singh Jutley G , Looms J , Ford L . N‐desethyl isotonitazene detected in polydrug users admitted to hospital in Birmingham, United Kingdom. Clin Toxicol [Internet]. 2024;62(1):19–25. 10.1080/15563650.2024.2309321 38353737

[17] Amaducci A , Aldy K , Campleman SL , Li S , Meyn A , Abston S , et al. Naloxone use in novel potent opioid and fentanyl overdoses in emergency department patients. JAMA Netw Open. 2023;6(8):e2331264. 10.1001/jamanetworkopen.2023.31264 37642962 PMC10466160

[18] Corporation AP . ZIMHI (naloxone hydrochloride injection). 2021.

[19] Inc. HSU . KLOXXADO (naloxone hydrochloride) nasal spray [prescribing information]. 2021.

[20] Shafi A , Berry AJ , Sumnall H , Wood DM , Tracy DK . Synthetic opioids: a review and clinical update. Ther Adv Psychopharmacol. 2022;12:1–16. 10.1177/20451253221139616 PMC9747888 36532866

[21] Rzasa Lynn R , Galinkin JL . Naloxone dosage for opioid reversal: current evidence and clinical implications. Ther Adv Drug Saf. 2018;9(1):63–88. 10.1177/2042098617744161 29318006 PMC5753997

[22] Adapt Pharma Inc . NARCAN (naloxone hydrochloride) nasal spray [prescribing information]. 2015.

[23] Parsons G . Nyxoid (naloxone) nasal spray for opioid overdose. Prescriber. 2019;30(5):38–39. 10.1002/psb.1764

[24] Torralva R , Janowsky A . Noradrenergic mechanisms in fentanyl‐mediated rapid death explain failure of naloxone in the opioid crisis. J Pharmacol Exp Therapeut. 2019;371(2):453–475. 10.1124/jpet.119.258566 PMC6863461 31492824

[25] Bird HE , Huhn AS , Dunn KE . Fentanyl absorption, distribution, metabolism, and excretion: narrative review and clinical significance related to illicitly manufactured fentanyl. J Addict Med. 2023;17(5):503–508. 10.1097/ADM.0000000000001185 37788600 PMC10593981

[26] Moss RB , Carlo DJ . Higher doses of naloxone are needed in the synthetic opiod era. Subst Abuse Treat Prev Policy. 2019;14(1):1–6. 30777088 10.1186/s13011-019-0195-4 PMC6379922

[27] Levine R , Veliz S , Singer D . Wooden chest syndrome: beware of opioid antagonists, not just agonists. Am J Emerg Med. 2020;38(2):411.e5–411.e6. 10.1016/j.ajem.2019.09.009 31831344

[28] Rosal NR , Thelmo FL , Tzarnas S , DiCalvo L , Tariq S , Grossman C . Wooden chest syndrome: a case report of fentanyl‐induced Chest Wall rigidity. J Investig Med High Impact Case Rep. 2021;9:0–2. 10.1177/23247096211034036 PMC8312149 34301155

[29] Moss RB , McCabe Pryor M , Baillie R , Kudrycki K , Friedrich C , Reed M , et al. Higher naloxone dosing in a quantitative systems pharmacology model that predicts naloxone‐fentanyl competition at the opioid mu receptor level. PLoS ONE. 2020;15(6):1–13. 10.1371/journal.pone.0234683 PMC7297366 32544184

[30] Mann J , Samieegohar M , Chaturbedi A , Zirkle J , Han X , Ahmadi SF , et al. Development of a translational model to assess the impact of opioid overdose and naloxone dosing on respiratory depression and cardiac arrest. Clin Pharmacol Ther. 2022;112(5):1020–1032. 10.1002/cpt.2696 35766413

[31] Carpenter J , Murray BP , Atti S , Moran TP , Yancey A , Morgan B . Naloxone dosing after opioid overdose in the era of illicitly manufactured fentanyl. J Med Toxicol. 2020;16(1):41–48. 10.1007/s13181-019-00735-w 31471760 PMC6942078

[32] Cantwell K , Dietze P , Flander L . The relationship between naloxone dose and key patient variables in the treatment of non‐fatal heroin overdose in the prehospital setting. Resuscitation. 2005;65(3):315–319. 10.1016/j.resuscitation.2004.12.012 15919568

[33] Bell A , Bennett AS , Jones TS , Doe‐Simkins M , Williams LD . Amount of naloxone used to reverse opioid overdoses outside of medical practice in a city with increasing illicitly manufactured fentanyl in illicit drug supply. Subst Abus. 2019;40(1):52–55. 10.1080/08897077.2018.1449053 29558283

[34] Rock P , Slavova S , Westgate PM , Nakamura A , Walsh SL . Examination of naloxone dosing patterns for opioid overdose by emergency medical services in Kentucky during increased fentanyl use from 2018 to 2021. Drug Alcohol Depend. 2024;255:1–17. 10.1016/j.drugalcdep.2023.111062 PMC11057324 38157702

[35] Somerville NJ , O'Donnell J , Gladden RM , Zibbell JE , Green TC , Younkin M , et al. Characteristics of fentanyl overdose — Massachusetts, 2014–2016. MMWR Morb Mortal Wkly Rep. 2017;66(14):382–386. 10.15585/mmwr.mm6614a2 28406883 PMC5657806

[36] Lemen PM , Garrett DP , Thompson E , Aho M , Vasquez C , Park JN . High‐dose naloxone formulations are not as essential as we thought. Harm Reduct J. 2024;21(1):1–17. 38741224 10.1186/s12954-024-00994-z PMC11089786

[37] Neale J , Strang J . Naloxone‐does over‐antagonism matter? Evidence of iatrogenic harm after emergency treatment of heroin/opioid overdose. Addiction. 2015;110(10):1644–1652. 10.1111/add.13027 26119038

[38] Strang J , Neale J , McDonald R , Kalk N . Toxicity: exploring and expanding the concept. Addiction. 2018;113(4):592–594. 10.1111/add.14080 29205614

[39] Pergolizzi JV , Dahan A , Ann LeQuang J , Raffa RB . Overdoses due to fentanyl and its analogues (F/FAs) push naloxone to the limit. J Clin Pharm Ther. 2021;46(6):1501–1504. 10.1111/jcpt.13462 34111307

[40] Payne ER , Stancliff S , Rowe K , Christie JA , Dailey MW . Comparison of administration of 8‐milligram and 4‐milligram intranasal naloxone by law enforcement during response to suspected opioid overdose — New York, march 2022–august 2023. MMWR Morb Mortal Wkly Rep. 2024;73(5):110–113. 38329911 10.15585/mmwr.mm7305a4 PMC10861201

[41] Farkas A , Lynch MJ , Westover R , Giles J , Siripong N , Nalatwad A , et al. Pulmonary complications of opioid overdose treated with naloxone. Ann Emerg Med. 2020;75(1):39–48. 10.1016/j.annemergmed.2019.04.006 31182316

[42] Kummer RL , Kempainen RR , Olives TD , Leatherman JW , Prekker ME . Naloxone‐associated pulmonary edema following recreational opioid overdose: a case series. Am J Emerg Med. 2022;53:41–43. 10.1016/j.ajem.2021.12.030 34973491

[43] Ferguson N , Farrugia A , Moore D , Fraser S . Remaking the ‘angry Narcanned subject’: affording new subject positions through take‐home naloxone training. Int J Drug Policy. 2024;123(November 2023):104253. 10.1016/j.drugpo.2023.104253 37995551

[44] Scheuermeyer FX , DeWitt C , Christenson J , Grunau B , Kestler A , Grafstein E , et al. Safety of a brief emergency department observation protocol for patients with presumed fentanyl overdose. Ann Emerg Med [Internet]. 2018;72(1):1–8.e1. 10.1016/j.annemergmed.2018.01.054 29530654

[45] Neale J , Kalk NJ , Parkin S , Brown C , Brandt L , Campbell ANC , et al. Factors associated with withdrawal symptoms and anger among people resuscitated from an opioid overdose by take‐home naloxone: exploratory mixed methods analysis. J Subst Abuse Treat [Internet]. 2020;117(March):108099. 10.1016/j.jsat.2020.108099 32811629 PMC7491601

[46] Bessen S , Metcalf SA , Saunders EC , Moore SK , Meier A , McLeman B , et al. Barriers to naloxone use and acceptance among opioid users, first responders, and emergency department providers in New Hampshire, USA. Int J Drug Policy [Internet]. 2019;74:144–151. Available from: http://europepmc.org/abstract/MED/31590090. 10.1016/j.drugpo.2019.09.008 31590090 PMC7153573

[47] Strickland JC , Marks KR , Smith KE , Ellis JD , Hobelmann JG , Huhn AS . Patient perceptions of higher‐dose naloxone nasal spray for opioid overdose. Int J Drug Policy. 2022;106:1–10. 10.1016/j.drugpo.2022.103751 PMC9378440 35636070

[48] Dietze P , Jauncey M , Salmon A , Mohebbi M , Latimer J , Van Beek I , et al. Effect of intranasal vs intramuscular naloxone on opioid overdose: a randomized clinical trial. JAMA Netw Open. 2019;2(11):1–12. 10.1001/jamanetworkopen.2019.14977 PMC6902775 31722024

[49] Dahan A , Franko TS , Carroll JW , Craig DS , Crow C , Galinkin JL , et al. Fact vs. fiction: naloxone in the treatment of opioid‐induced respiratory depression in the current era of synthetic opioids. Front. Public Health. 2024;12:12. 10.3389/fpubh.2024.1346109 PMC10933112 38481848

[50] Russell E , Hawk M , Neale J , Bennett AS , Davis C , Hill LG , et al. A call for compassionate opioid overdose response. Int J Drug Policy. 2024;133(September):104587. 10.1016/j.drugpo.2024.104587 39299143

[51] Tylleskar I , Gjersing L , Bjørnsen LP , Braarud AC , Heyerdahl F , Dale O , et al. Prehospital naloxone administration ‐ what influences choice of dose and route of administration? BMC Emerg Med. 2020;20(1):1–10. 32891142 10.1186/s12873-020-00366-3 PMC7487505

[52] Kelly AM , Kerr D , Dietze P , Patrick I , Walker T , Koutsogiannis Z . Randomised trial of intranasal versus intramuscular naloxone in prehospital treatment for suspected opioid overdose. Med J Austr. 2005;182(1):24–27. 10.5694/j.1326-5377.2005.tb06550.x 15651944

[53] Kerr D , Kelly AM , Dietze P , Jolley D , Barger B . Randomized controlled trial comparing the effectiveness and safety of intranasal and intramuscular naloxone for the treatment of suspected heroin overdose. Addiction. 2009;104(12):2067–2074. 19922572 10.1111/j.1360-0443.2009.02724.x

[54] Skulberg AK , Tylleskär I , Valberg M , Braarud AC , Dale J , Heyerdahl F , et al. Comparison of intranasal and intramuscular naloxone in opioid overdoses managed by ambulance staff: a double‐dummy, randomised, controlled trial. Addiction. 2022;117(6):1658–1667. 10.1111/add.15806 35137493 PMC9302677

[55] Cho R , Purssell R , Joe R , Wang YE , O'Sullivan F , Lin K , et al. Opioid overdose and naloxone dosing at Insite supervised injection Facility in British Columbia: a retrospective cohort study. Can J Addict. 2022;13(4):22–31.

[56] Moss RB , McCabe Pryor M , Baillie R , Kudrycki K , Friedrich C , Reed M , et al. Higher naloxone dosing in a quantitative systems pharmacology model that predicts naloxone‐fentanyl competition at the opioid mu receptor level. PLoS One [Internet]. 2020;15(6):1–13. 10.1371/journal.pone.0234683 PMC7297366 32544184

[57] Lavonas EJ , Akpunonu PD , Arens AM , Babu KM , Cao D , Hoffman RS , et al. 2023 American Heart Association focused update on the Management of Patients with cardiac arrest or life‐threatening toxicity due to poisoning: an update to the American Heart Association guidelines for cardiopulmonary resuscitation and emergency Cardiov. Circulation. 2023;148(16):e149–e184. 10.1161/CIR.0000000000001161 37721023

[58] Ellison M , Hutton E , Webster L , Skolnick P . Reversal of opioid‐induced respiratory depression in healthy volunteers: comparison of intranasal Nalmefene and intranasal naloxone. J Clin Pharmacol. 2024;64(7):828–839. 10.1002/jcph.2421 38436495

[59] Cipriano A , Apseloff G , Kapil RP , He E , Shet M , Harris SC . Time course of reversal of fentanyl‐induced respiratory depression in healthy subjects by intramuscular Nalmefene and intramuscular and intranasal naloxone. J Clin Pharmacol. 2024;65(2):206–216. 10.1002/jcph.6132 39347921 PMC11771590

[60] Infante AF , Elmes AT , Gimbar RP , Messmer SE , Neeb C , Jarrett JB . Stronger, longer, better opioid antagonists? Nalmefene is NOT a naloxone replacement. Int J Drug Policy. 2024;124(January):104323. 10.1016/j.drugpo.2024.104323 38232438

[61] Rosenberg M , Chai G , Mehta S , Schick A . Trends and economic drivers for United States naloxone pricing, January 2006 to February 2017. Addict Behav. 2018;86:86–89. 10.1016/j.addbeh.2018.05.006 29914719 PMC6261609

[62] Madah‐Amiri D , Clausen T , Lobmaier P . Rapid widespread distribution of intranasal naloxone for overdose prevention. Drug Alcohol Depend [Internet]. 2017;173:17–23. 10.1016/j.drugalcdep.2016.12.013 28182982

[63] Chen Y , Wang Y , Nielsen S , Kuhn L , Lam T . A systematic review of opioid overdose interventions delivered within emergency departments. Drug Alcohol Depend [Internet]. 2020;213(April):108009. 10.1016/j.drugalcdep.2020.108009 32580113

[64] Wagner KD , Oman RF , Smith KP , Harding RW , Dawkins AD , Lu M , et al. “Another tool for the tool box? I'll take it!”: feasibility and acceptability of mobile recovery outreach teams (MROT) for opioid overdose patients in the emergency room. J Subst Abuse Treat [Internet]. 2020;108(January 2019):95–103. 10.1016/j.jsat.2019.04.011 31079951

[65] Lowenstein M , Sangha HK , Spadaro A , Perrone J , Delgado MK , Agarwal AK . Patient perspectives on naloxone receipt in the emergency department: a qualitative exploration. Harm Reduct J [Internet]. 2022;19(1):97. 10.1186/s12954-022-00677-7 36028882 PMC9412772

